# Medical Society of the County of Erie

**Published:** 1900-08

**Authors:** Franklin C. Gram


### Society Proceedings.

### MEDICAL SOCIETY OF THE COUNTY OF ERIE.

*Semi-annual Meeting, June 12, 1900.*

Reported by FRANKLIN C. GRAM, M. D., Secretary.

DR. E. H. BALLOU, the president, called the semi-annual meeting to order shortly after 10 a.m., June 12, 1900, in the Y.M.C.A. parlors. After the minutes of the previous meetings had been adopted the chair appointed Drs. Ingraham, Strong and W. W. Potter a committee to prepare a suitable memorial on the death of Dr. E. T. Dorland. This committee later presented the following, which was adopted:

### IN MEMORIAM—E. T. DORLAND, M. D.

Again the Medical Society of Erie County is called upon to mourn the loss of one of its oldest and most distinguished members, Dr. Elias T. Dorland, and it desires to enter upon its records its appreciation of his professional worth and character.

Dr. Dorland was born in Union, Dutchess County, New York, April 12, 1832. His father, Joseph Dorland was a prominent physician in that county. In early life, he came with his father to East Hamburg—now Orchard Park—this county, and received his preliminary education in the public school of this village, and at Springville Academy. He began his medical studies in Buffalo, and attended the Medical Department of the University for one year. Later, he became a student of the University of Michigan, graduating from this institution in 1854. For two years he was a resident of the Erie County Almshouse when he located at Lagrange, Duchess County, where he remained twelve years. He then returned to East Hamburg, the home of his childhood. In 1872 he came to Buffalo, where he since resided, and continued in the practice of his chosen profession until three years ago, when failing health compelled him to retire from active work, yet he always maintained a deep interest in the profession and was a loyal disciple of legitimate medicine. Dr. Dorland was President of this Society in 1886. He was one of the original members of the New York State Medical Association. He was endowed with one quality which it would be wise for all the younger members of the profession to emulate—his business ability. He was always kind and genial, and possessed of an amount of wit and humor far beyond the average.

Dr. Dorland was one of the best known and most respected, of the physicians of Buffalo, and his death leaves a sadly deplored vacancy in our ranks.

HENRY D. INGRAHAM,
O. C. STRONG,
WILLIAM WARREN POTTER.

Dr. W. W. Potter moved the adoption of the amendments to the by-laws, as presented at the January meeting. The motion was carried.

Dr. W. W. Potter, chairman of the committee on membership, then presented the following names of candidates who desired admission to the society: Drs. Chauncey P. Smith, William R. Little, Robert K. Grove and Francis M. O'Gorman. The membership committee reported favorably and they were elected.

Dr. J. B. Coakley submitted the following report from the Board of Censors, which was adopted:

*To the Medical Society of the County of Erie:*

*Gentlemen*—Your Board of Censors hereby makes report of its work and transactions since your last annual meeting, as follows:

During the past year your board has continued to employ the services of Hon. Tracy C. Becker as counsel for this society at the same compensation as was paid to him during the preceding year, and all complaints needing legal prosecution have been referred to him therefor.

During the past half year complaints have been made against certain persons engaged in business and advertising themselves as dermatologists, chiropodists, etc., and using the title of "Dr." in connection therewith without being properly licensed and registered under the medical laws of this state. Such persons have been notified that they were violating the law and have, under threat of persecution therefor, discontinued the use of such title.

In the case of William Carpenter an indictment has been found for practising in this city without a license and said case is still pending and will probably be tried at the next criminal term of the Supreme Court.

About the 1st of April, 1900, complaint was made to your board that one Frances Andrus, who advertised herself as "the person who discovered the process of permanently removing freckles, moles, birthmarks and all blemishes from the human skin," had treated the young child of a Mrs. Kendall, by applying some lotion or substance to a face-blemish or mother's mark on the face of said child, and had permanently disfigured the child's face without removing the blemish or mark. On examination of the child by some of the members of your board and by Dr. Ernest Wende, this complaint seemed to be substantiated by the fact that the child's face bore evidences of scars in and around said birthmark and blemish, which were probably caused by the application of some acid or other substance. In addition to this Mrs. Kendall produced a written contract, signed by Mrs. Andrus, guaranteeing for a sum stated to remove said birthmark and blemish. Our counsel procured a warrant to be issued by the police magistrate for the arrest of Mrs. Andrus, an examination was had and she was held to answer before the Grand Jury, and in default of $500 bail was committed to the County Jail. The Grand Jury of the County Court indicted her for the misdemeanor of practising medicine without a license, and on the 10th and 11th of April she was tried and acquitted by verdict of the jury of the misdemeanor charged, although the proof was absolute and undenied that she had made the contract with Mrs. Kendall and had given the treatment to the child as above mentioned.

The case seems to have been faithfully prosecuted by the District-Attorney, and the verdict to be one of those miscarriages of justice which will sometimes occur in a great city like this, in which a jury may be impanneled consisting of men who are hostile to the execution of the laws designed for the protection of themselves and their families, as well as other citizens, against the illegal practice of medicine.

In this case, and in the case of numerous complaints made to us in reference to the practices of so-called osteopaths, masseurs, and persons who advertise to cure everything by the use of the electric current and other similar devices, although not advertising or using the title of "Dr.," . . . we are constantly confronted by the limitation placed upon the meaning of the terms "medical or surgical treatment" used in the statute against the illegal practice of medicine, by the decision of Judge Daniels in the old case of Smith against Lane (24 Hun., 32) in which it was held at the General Term of the Supreme Court that the practice of medicine in the meaning of the law was practically restricted to the prescribing of drugs or using surgical methods of cure, thus easily permitting unchallenged the various socalled systems, as osteopathy, massage and the like.

We are informed by our counsel that after a conference by him with Dr. Maurice J. Lewi of the State Board of Medical Examiners in New York and a meeting of the full board of examiners, together with the Secretary of the Board of Regents and the Statutory Revision

Commissioners at Albany, and correspondence with Hon. Robert S. Taylor, counsel for the New York County Medical Society concerning this defect in the medical law, and other defects therein in reference to the revocation of licenses, etc., several amendments to the medical law were prepared and approved by the authorship, and introduced as part of the scheme of revision of the statutes by the Statutory Revision Commission, at the last legislature. There was no opposition to these amendments, but for reasons connected with the abolition of the Statutory Revision Commission, and the appointment of a committee of the legislature to do this work, none of the bills prepared and presented by the Statutory Revision Commission were passed this year. We are, however, assured that the proposed amendments will be pressed upon the attention of the next legislature by the State Board of Medical Examiners and the Regents of the University, as well as by our counsel, and the counsel for the New York County Medical Society, who are all acting together, with more than a fair chance of such amendments being adopted.

Respectfully submitted,

J. B. Coakley, Chairman.
H. R. Hopkins.
Thomas F. Dwyer.
Irving W. Potter.

Dr. A. L. Benedict stated as there were two medical societies in this state, and as most of the members belonged to both, it might be well for this society to inaugurate such steps as will ultimately bring about a union. He therefore submitted the following resolution:

*Resolved*, That the president appoint a committee of three to consider the feasibility of unifying the dual system of state and county medical organisations existing in the State of New York, and of securing representation in the American Medical Association, said committee to report at the annual meeting in January, 1901.

Dr. Eli H. Long said he was not aware of any second county organisation at the present time.

Dr. William Warren Potter said he considered the resolution unnecessary, to say the least. There were rumors of the organisation of an Erie County Association, but until this society received official notice to that effect he was opposed to meddling with other bodies. This society received its power from the State Society, in existence since 1806, and was responsible to it for any action it might take in the premises.

Dr. J. B. Coakley favored the resolution and seconded it. He considered it in proper spirit and hoped to see the time when there will be but one county, state and national organization. This is the only state in which two such organisations exist. He never affiliated with the second organisation because he was opposed to such friction, but he favored a union.

Dr. Pohlman.—"As I understand it a man can not belong to both, except he makes a liar of himself?"

Dr. W. W. Potter.—"You would not in all probability be recognised by the association as a delegate from this society, but you might attend the national association as an individual."

Dr. Geo. F. Cott.—"This society is the only one recognised by law in this state."

The resolution was adopted and the president appointed the following committee to consider its provisions: Dr. A. L. Benedict, chairman; Coakley, Thornton, E. H. Long and W. W. Potter.

Dr. C. E. Congdon called attention to several conditions at present existing in Buffalo to the detriment of legal practitioners. Inasmuch as no improvement of the evils could be expected through the District Attorney's office, he moved that the society employ a competent attorney with bull-dog tenacity, who should look after all illegal practitioners, and who should receive as his fees all the fines to which this society is entitled by law, the appointment of this attorney to be left with the president.

Dr. Walsh seconded the resolution and approved its sentiment.

Dr. E. H. Long raised the point of order that the resolution was in conflict with the by-laws of this society, and should properly be offered as an amendment to the same.

The president sustained the point of order, but as Dr. Congdon was suddenly called away the subject was dropped.

The secretary stated that the parlors of the Y.M.C.A. had been placed at the free disposal of this society. Several meetings had already been held there and he moved that in addition to the usual vote of thanks the janitor be given $5 for additional work. Carried.

Dr. Geo. F. Cott then explained the treatment of radical operations on the ear and exhibited several cases.

Dr. F. C. Gram read a paper on The Recent Epidemic of Measles in Buffalo, and gave figures and results which he had collected as Registrar of Vital Statistics. (See p. 39.)

Prof. C. N. Millard, supervisor of Grammar Grades of Public Schools, discussed the paper, giving the relation which the epidemic had to the public schools. He said that the statistics given hardly represented the black eye which the schools received and that it would take a long time to recover from the effects. In school No. 56, with an average attendance of 600, there were 89 out in one week, one class of 47 was decreased to 22. In No. 59, with an average attendance of 628, there were 427 out for measles in one week. In No. 44, with a registration of 977, there were 252 out in one week of which 103 were from the first grade in which the registration was 260. No. 26 lost 300 out of 720 in one week. No. 59 had its average cut in two by measles. He believed that in most cases the danger from contagion was done before it was discovered by the principal, and questioned whether exclusion from school had the desired effect when the children mingle in play on the streets or at home, ride in street cars, attend church and the like. In some cases of truancy the children gave measles as an excuse. He hoped that additional health inspectors would be appointed to alleviate some of the present causes for complaint.

Dr. IRVING M. SNOW said the recent epidemic of measles was the severest he had ever witnessed. The statistics presented by Dr. Gram showed that with the closing of the schools during vacation the number of cases immediately dropped to a low figure, and immediately increased when schools were reopened. Closed rooms, at a season when windows could not be freely opened, also contributed in the spread. He questioned the mortality statistics.

Dr. H. D. INGRAHAM believed the mortality rate was greater than reported, otherwise it must have been considerably below the average.

In one New York hospital it was 70 per cent. and Dr. Jacobi holds the mortality rate of epidemics at from 25 to 33 per cent. The mortality is greatest in children under two years.

Little can be done to prevent the spread. The only effective way is to put the child in bed as soon as it shows symptoms of measles and keep it there until it has completely recovered.

Dr. E. H. LONG considered that it would be valuable to the school department and physicians if both joined in discussion of similar subjects oftener. The Health Department was likewise a great educator of the public, which clung to erroneous ideas concerning many medical subjects. He believed the important point in the statistics presented was indicated by the mortality and the season. He also briefly discussed the treatment.

At 12.30 the society adjourned.

